# Biological relevance of cell-in-cell in cancers

**DOI:** 10.1042/BST20180618

**Published:** 2019-03-08

**Authors:** Hannah L. Mackay, Patricia A.J. Muller

**Affiliations:** 1 Institute of Cancer and Genomic Sciences, University of Birmingham, Birmingham B15 2TT, U.K.; 2 Cancer Research UK Manchester Institute, The University of Manchester, Alderley Park, U.K.

**Keywords:** biomarkers, cancer, cannibalism, cell-in-cell, entosis, p53

## Abstract

Cell-in-cell (CIC) is a term used to describe the presence of one, usually living, cell inside another cell that is typically considered non-phagocytic. Examples of this include tumour cells inside tumour cells (homotypic), mesenchymal stem cells inside tumour cells (heterotypic) or immune cells inside tumour cells (heterotypic). CIC formation can occur in cell lines and in tissues and it has been most frequently observed during inflammation and in cancers. Over the past 10 years, many researchers have studied CIC structures and a few different models have been proposed through which they can be formed, including entosis, cannibalism and emperipolesis among others. Recently, our laboratory discovered a role for mutant p53 in facilitating the formation of CIC and promoting genomic instability. These data and research by many others have uncovered a variety of molecules involved in CIC formation and have started to give us an idea of why they are formed and how they could contribute to oncogenic processes. In this perspective, we summarise current literature and speculate on the role of CIC in cancer biology.

## Introduction

**Figure 1.**
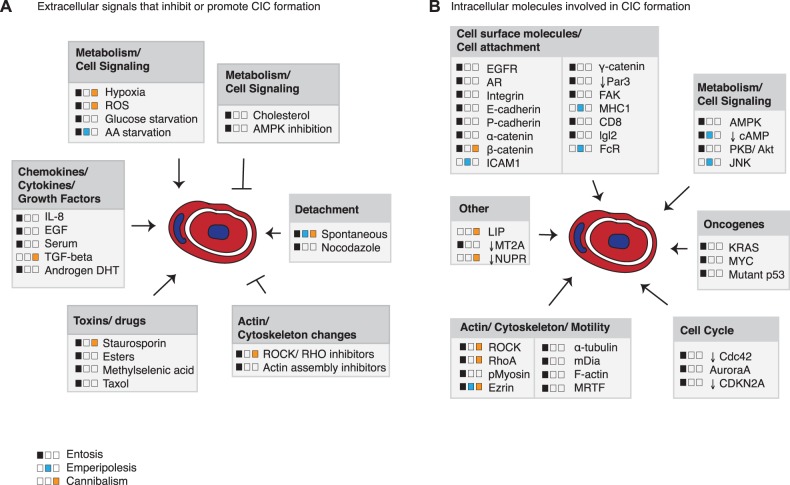
Signals and molecules involved in CIC. (**A**) Extracellular signals regulating CIC formation through entosis, cannibalism or emperipolesis as indicated in the legend. No differentiation was made between homotypic or heterotypic cannibalism. (**B**) Intracellular molecules involved in entosis, cannibalism or emperipolesis as indicated in the legend. No differentiation was made between homotypic or heterotypic cannibalism.

**Figure 2.**
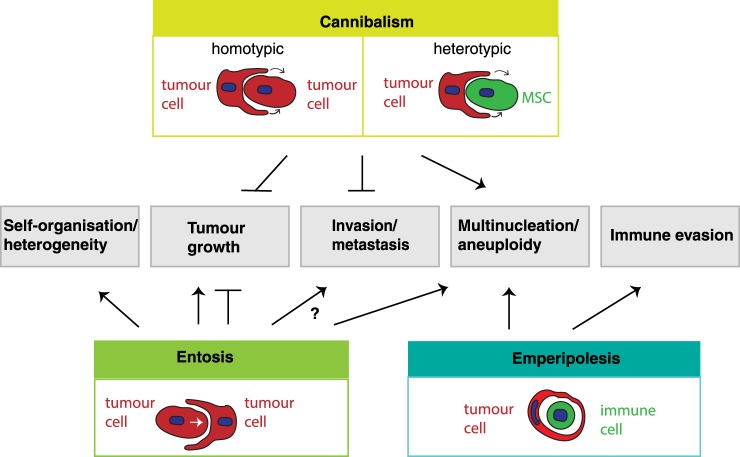
The role of different CIC formations in different aspects of cancer biology. Emperipolesis is only depicted as an immune cell residing inside a tumour cell, although this term has been used for other engulfment processes and other outcomes, which are not included in this figure.

Cell-in-cell (CIC) is a term used in histopathology to describe a phenomenon in which one whole cell can be detected inside another cell [1,2]. The inside cell is often rounded up, usually alive, and resides inside a large vacuole in the external cell. The external cell frequently appears enlarged with a squashed half-moon-shaped nucleus but can have multiple nuclei. Across a variety of cancers, these structures have been seen for decades in tissue samples, but only more recently has this phenomenon been studied in more detail *in vitro*. Many have defined the triggers resulting in increased or reduced numbers of CIC structures (Figure 1A). A few studies have investigated how they are formed and which molecules are involved (Figure 1B), but arguably the most intriguing and elusive question at this moment in time is what their role in cancers is (Figure 2). In general, CIC numbers are the highest in the most aggressive cancers, suggesting a pro-tumorigenic role.

CIC can be formed through different processes and can constitute two or more cells from the same origin (e.g. two tumour cells, homotypic) or cells from different origins (e.g. immune cells inside tumour cells, heterotypic). Many different terms are in circulation to describe the formation of CICs, which include but are not limited to entosis, engulfment, emperipolesis and cannibalism (recently defined in [2]). The differences between the types of CIC formation can vary in terms of the cell types involved, which cell drives the formation of the CIC structure and the mechanisms underlying the process. Although *in vitro* testing can differentiate some characteristics, to confirm which process leads to CIC *in vivo* can be quite challenging, if possible at all. Some processes have been described in more detail than others and phenotypical characteristics used to define one type of formation are now found to play a role in other types of CIC formation. In this perspective, we will review the literature on CIC formation in cell lines, in cancers and under unperturbed physiological conditions and we will discuss the potential of CIC as a biomarker for disease stage in cancers. We will use the nomenclature for each CIC formation event as used by the authors, although insufficient data to conclude which CIC formation process underlies the observed CIC structure could have resulted in inconsistent terminology.

## The formation of CIC structures

Many signals and intracellular proteins have been implicated in the different types of CIC formation (Figure 1). In entosis, the cell that is ultimately internalised is actively driving entosis [3]. This process is, therefore, also referred to as in-cell invasion and most often leads to the death of the internal cell. A low level of entosis is encountered in susceptible cell lines under normal tissue culture conditions, but higher rates are seen when cells are grown in matrix-detached conditions [3–5]. Even in ‘spontaneous’ entosis under normal growth conditions, the invading cell detaches prior to engulfment, suggesting that matrix detachment is an important trigger for entosis [6,7]. Under normal culture conditions, matrix detachment occurs prior to mitosis or apoptosis [6,8]. Wang et al. [8] described that cells that are inherently incapable of apoptosis are likely to invade into neighbours upon apoptotic triggers. These data suggest that entosis represents a safety mechanism to remove ‘abnormal’, detached cells from a tissue. Other activators of entosis include reactive oxygen species, methylselenoesters, epidermal growth factor, IL-8 and serum [8–13] (Figure 1A), some of which might trigger entosis simply by causing mitosis or apoptosis.

A prerequisite for entosis is an interaction between the two cells, which is mediated through the catenin and cadherin adhesion molecules [3,13,14]. To form a CIC structure, the driver cell needs to be relatively rigid, whereas the external cell requires high deformability to extend its membrane all the way around the invading cell [15]. The rigidity of the driver cells is mediated through changes in the actin cytoskeleton (e.g. actinomyosin), driven by the Rho/ROCK or DIA pathway [3,6,15–18]. In response to this tension, the transcription factor MRTF (myocardin-related transcription factor) enhanced the expression of Ezrin, which was shown to be required for the actual invasion into the host cell [18].

Entosis is thought to be an energy-efficient process. The rigid driver cell invading into the deformable external cell can be compared with a stone hitting a soft pillow. By sheer motion, the rigid cell will end up mostly engulfed in the deformable external cell, to which it is immediately anchored through adhesion molecules working like velcro. The external cell then only needs to close up its membranes in order to engulf the driver cell. Entosis could, therefore, be a means for cells that are lowest in energy and nutrients to sacrifice themselves to less starved neighbouring cells, possibly ensuring the maintenance of the population and structural tissue integrity. This hypothesis is supported by the notion that entosis is induced upon starving cells of nutrients and energy and that glucose-deprived cells can survive by living off their neighbours [19,20].

Studies specifically addressing the mechanisms underlying cannibalism are more scarce than equivalent studies for entosis. In cannibalism, the driver cell is the external cell that extends protrusions around the engulfed cell, which would, therefore, be less energy-efficient than entosis. Cannibalism is thought to resemble phagocytosis and loss of Nupr1 increased the expression of genes related to phagocytosis and the actin cytoskeleton. In contrast with entosis, knockdown of E-cadherin could not inhibit cannibalism and disruption of the ROCK signalling pathway actually enhanced pancreatic cell cannibalism [21]. Heterotypic forms of cannibalism have also been described in cancer cells. MDA-MB-231 tumour cells cannibalised mesenchymal stem cells, which were ROCK-dependent and correlated with increased expression of JNK and secretion of specific cytokines [22,23].

The term cannibalism has also been used to describe melanoma tumour cells engulfing immune cells [2,24]. The more widely used term for tumour cells engulfing immune cells is emperipolesis, although this term has also been used for other non-tumourous CIC structures, including oligodendrocytes residing in astrocytes or erythrocytes residing in Kupffer cells [25–28]. Tumour cell-associated emperipolesis has mainly been studied in histological sections with limited mechanistic details. Tumour cells can engulf lymphocytes or natural killer cells, followed by apoptotic degradation of these cells [29]. This is termed emperitosis after emperipolesis and apoptosis. Emperitosis would prevent autorecognition of the tumour cells and evasion of immune response and requires actin cytoskeleton changes and Ezrin regulation [24]. Chen et al. [30] determined that various tumour cells which engulf neighbouring tumour cells are more likely to also engulf immune cells. No differentiation was made between entosis or cannibalism, but the present study does suggest that similarities between homotypic CIC formation and emperipolesis could exist. In contrast, Cano et al. [21] found that pancreatic tumour cells cannibalised living lymphocytes in a different manner than neighbouring tumour cells, which would favour the idea that cannibalism and emperipolesis are different processes.

## Cellular consequences of CIC

A commonality between all the different types of CIC formation is the cellular outcome of CIC for which one has to consider the fate of the internal and the external cells. For engulfment, entosis and for emperipolesis, it has been described that the internal cell can die, divide or escape [2]. The frequency at which each of these events occurs seems dependent on characteristics of both the internal and the external cell and perhaps not so much on the process of CIC formation. Impairment of autophagy has been shown to decrease the likelihood of the internal cell being digested, leading to release or escape of the internal cell [31]. In response to engulfment, LC3 is recruited to the host vacuole enclosing the internalised cell. This caused fusion with lysosomes leading to acidification of the vesicle and degradation of the internal cell via lysosomal enzyme degradation mediated by FAK signalling in an apoptotic-dependent and -independent manner [4,6,8,31,32]. Most likely, the internal cell's intrinsic ability to withstand the acidic environment also plays a role in which type of death occurs and whether or not the cell is able to escape.

The external cell can remain unaffected, die or undergo replication stress leading to chromosomal rearrangements and/or multinucleation [10,30,33,34]. Our recent work showed that the p53 status of external cells is a determinant as to whether the external cell survives or not [9]. In 50% of all cancers, *Tp53* is mutated leading to loss of p53 expression or the expression of a mutant p53 protein. Mutant p53 proteins have been shown to not only lose wild-type (WT) function but also gain oncogenic traits such as invasion and metastasis [35,36]. We demonstrated that the expression of mutant p53, but not the loss of p53 or expression of WT p53, allows external cells to survive the burden of harbouring a cell. External cells showed signs of replication stress and repeatedly failed to divide leading to the formation of multinucleated cells with internal cells. On the contrary, WT p53 cells or p53 null cells more often died upon cell division suggesting that mutant p53 status allows for replication stress survival instigated by internalised cells. Cell death of external cells was also seen in MDA-MB-231 cells cannibalising human umbilical mesenchymal stem cells [22] and in tumour cells that were penetrated by HOTOZ cells (a lymphocyte-like cell line) through emperipolesis [37]. It will be interesting to determine if other stem cells can similarly suppress the growth of tumour cells and what characteristics of the external and internal cells determine the fate of the external cells.

The fact that starvation can also induce CIC formation suggests that CIC could cause a survival advantage for the external cell. This was seen for entosis upon glucose deprivation and amino acid starvation [19,20,32]. Increased AMPK levels in the invading driver cell as a result of glucose starvation promoted entosis in a variety of breast cancer cells [20]. Under amino acid starvation, PIKfyve was shown to induce shrinkage of the entotic vacuole to which the mTORC1 complex was recruited in order to recover amino acids from the then digested cell. Cannibalistic melanoma cells were also able to survive amino acid starvation or serum starvation in a CIC-dependent manner in the presence, but not in the absence of lymphocytes [24]. These observations point to a survival advantage for the external cell irrespective of the type of CIC formation.

## Formation of CIC by non-cancerous cells

Although we are starting to learn more about the mechanisms underlying CIC and the triggers that can activate it, the reasons why cells start to form CIC structures are mostly unknown. Remarkably, some evidence of CIC structures in healthy normal tissue has been published, suggesting a physiological role for CIC formation. During embryogenesis, blastocyst cells were found to engulf luminal epithelial cells in a process required for embryo implantation [38]. Increased expression of β-catenin and the possibility to inhibit implantation by a ROCK inhibitor suggested that an entotic type of engulfment was underlying embryo implantation. In soft-shelled turtles, sertoli cells were found to engulf spermatozoa after hibernation [39] and CIC-like structures have been seen in the heart, involving cardiomyocytes [40]. However, *in vivo* studies of CIC are notoriously difficult for determining whether the inner cells are fully engulfed and through which type of CIC formation process the CIC structures are formed. Further studies are needed to assess this in more detail.

Emperipolesis is observed in normal and inflamed tissues. Erythrocytes reside in foetal liver Kupffer cells, possibly helping the maturation of these cells [28]. During normal and inflamed conditions, lymphocytes were seen within hepatocytes and in oligodendrocytes [41–44]. Ni et al. suggested that virus-infected lymphocytes could transmit certain viruses to otherwise non-susceptible epithelial cells through CIC formation and Benseler et al. suggested that emperipolesis of autoreactive T cells into hepatocytes is a way to maintain self-tolerance [44–46]. The term emperipolesis was also used to describe the engulfment of oligodendrocytes into astrocytes, which has been observed in cases of encephalitis, multiple sclerosis or Creutzfeldt–Jakob disease [25–27]. Wu and Raine [27] postulated that this behaviour could be a potential attempt to escape the harsh central nervous system environment in these conditions where severe inflammation and destruction were seen. It is evident that emperipolesis is involved in the immune response, but further research is needed to determine if it plays a role in dampening the immune response, protecting certain cells by creating a safe-haven or any other regulatory functions.

## CIC in tumour formation, maintenance and metastasis

The presence of CIC structures has been associated with advanced-stage cancers [9,33,47–49], suggesting a role for CIC in tumorigenesis and metastasis. Mouse studies in which CIC-forming cell lines were xenografted into recipient mice show the presence of CIC structures in tumours. However, functional consequences on the growth or metastatic potential of these tumours are often not seen or not described [6,9,16,50]. One reason for the lack of such studies is that inhibitors of entosis, cannibalism or emperipolesis are generically targeting many other processes such as motility, migration and metabolism, and so on. Our recent study in mice suggested that xenografted populations of engineered cells displaying high entotic activity were forming larger tumours than populations with lower entotic activity, suggesting a pro-tumorigenic role for entosis, but specific inhibitors are needed to confirm these results [9]. In contrast with entosis, tumour cell cannibalism decreased tumour growth. MDA-MB-231 cells that cannibalised mesenchymal stem cells entered dormancy or died [22,23].

To migrate and invade, tumour cells are known to become motile and deformable to allow them to migrate through narrow spaces in the microenvironment [51]. The molecular changes that are required for invasion can also be found in entotic cells, including a rearranged cytoskeleton, expression of Ezrin and increased ROCK activity (Figure 1B). It is, therefore, likely that similar mechanisms that allow for invasion allow for entosis, but the question as to whether entosis promotes invasion remains unanswered at this moment in time. The work by Cano et al. [21] suggests that cannibalism, which they found was ROCK and β-catenin independent coincided with an inability of cells to undergo TGF-β-induced EMT (epithelial to mesenchymal transition) changes required for metastasis. Together, these data suggest a pro-tumorigenic function for entosis and an anti-metastatic and anti-tumorigenic function for cannibalism (Figure 2), but further functional studies to confirm these speculations are necessary.

## The relationship between CIC structures and tumour promoters such as mutant p53

In our recent study, we investigated the role of mutant p53 in CIC formation and the functional consequences *in vivo*. *In vitro*, mixed populations of mutant p53 and p53 null cells displayed higher frequencies of CIC than p53 null or mutant p53 cells alone. In xenografts in mice, no growth advantage was found for mutant p53 cells compared with p53 null cells, but the mixed population of mutant p53 and p53 null cells had a growth advantage and the highest number of CIC structures [9]. Interestingly, in lung cancer tumours and in mouse xenografts, we noted that the more heterogenic a tumour was for (mutant) p53 expression, the more CIC structures were detected [9]. In addition, genomic instability was increased in lung tumours with a higher prevalence of CIC structures [9]. These data could mean that heterogeneity drives CIC formation, but also that CIC-associated replication stress triggers genomic alterations or multinucleations which favour tumour growth or metastasis.

Another tumour suppressor involved in entosis is *CDKN2A*. Inactivation of *CDKN2A* promoted entosis and *CDKN2A* expression and was inversely correlated to CIC formation in breast cancers [52]. Oncogenes have also been implicated in entosis and overexpression of *KRAS V12* or *c-Myc* was shown to promote the formation of the CIC structures [15,53]. *K-Ras V12* expression and loss of *CDKN2A* were found to down-regulate myosin and therefore cause deformability of the outer cell in the CIC structure. Whether or not *c-Myc* and mutant p53 cause deformability still remain to be elucidated. All these tumour suppressors and oncogenes are well known for their roles in tumour growth, invasion and metastasis. *c-Myc*, *KRAS V12* and mutant p53 expression as well as loss of *CDKN2A* are characteristics of the external and not internal cells of a CIC structure and are therefore likely to survive the entotic event [9,15,52,53]. In analogy to idea that cells involved in entosis have a ‘winner’ or ‘loser’ status based on cell deformability [15], entosis could therefore cause selection for those ‘winner’ tumour cells that have acquired mutations in *Myc*, *KRAS*, *CDKN2A* or *Tp53* and lead to heterogeneity that is seen within many tumours.

## CIC formation and drug resistance

The frequency with which internal cells die or escape can be highly variable. Overholtzer et al. [3] reported that the majority of internal cells died, but 18% in MCF10A and 12% in MCF7 escaped. Less than 50% of the H1299 cells died upon CIC formation and many H1299 and MDCK cells were able to escape CIC formation [9,34]. These studies raise the question whether there is an advantage for driver cells to invade into a neighbouring cell. Perhaps entosis could provide a safe environment in which a tumour cell might avoid both chemotoxic drug exposure and immune surveillance similar to oligodendrocytes sheltering in astrocytes upon inflammation and tissue damage [27]. The fact that certain chemotherapeutic drugs, including taxols [6] as well the pro-inflammatory signal IL-8 can promote CIC formation would favour this idea [13]. However, the invading driver cell does risk being killed and further evidence that tumour cells actively invade into other cells upon drug exposure is needed.

Protection against chemotherapy might also be achieved through heterotypic cannibalism. Bartosh et al. [23] demonstrated that heterotypic cannibalism led to dormancy in breast cancer cells. This could make cancer cells resistant to drug treatments that impact cytokinesis. Future research should focus on determining which chemotherapeutics or toxins induce homo- or heterotypic CIC formation, what CIC formation process is employed and what the functional and cellular consequences are.

## Future perspectives

Emperipolesis is used as a histopathological marker to diagnose Rosai–Dorfman disease in which immune cells are seen in phagocytic cells (histiocytes) [54]. Although homotypic CIC has been linked to advanced-stage cancers, it is not routinely used in the clinic for diagnostic or prognostic purposes. In lung, head and neck, and rectal cancers, increased numbers of homotypic CIC structures were correlated to reduced patient survival and increased disease recurrence [9,55,56]. Although performed in small numbers of patients, in some of these cancers, CIC was a better predictor than other histopathological characteristics for patient survival [9,56].

None of these studies have so far been large enough to show statistically significant correlations between chemotherapeutic treatments and the presence of CIC structures [21,56]. Many chemotherapeutics affect the cell cycle and it is, therefore, possible that chemotherapeutic treatment could influence CIC numbers. Interestingly, CIC structures have been found in effusion fluids and urine samples of patients with malignant disease [3,49]. Based on these data, CIC could represent an easily accessible biomarker for disease state and perhaps also for drug efficacy.

However, in pancreatic cancers, the presence of CIC correlated with decreased metastases [21]. These findings raise the question whether these differences are related to the different cancers in which they are found or whether the underlying processes through which CIC structures are formed are functionally different and are able to cause these different outcomes. Huang et al. [57] described the presence of four different subtypes of CIC structures based on immune cell and epithelial markers, which could differentiate between emperipolesis and homotypic CIC. It will be necessary to find more biomarkers that could distinguish between all types of CIC formation events to fully understand the role of CIC in cancer. Current biomarkers to differentiate the CIC formation processes are insufficient. ROCK inhibition was presumed entosis-specific, but heterotypic cannibalism could also be prevented by ROCK inhibition (Figure 1B). Similarly, β-catenin and Ezrin expression are not exclusive for entosis as they were shown involved in cannibalism and/or emperipolesis [3,16–18,20,24,29,58,59]. It can also not be excluded that homotypic CIC structures found in cancers are formed through a combination of entosis and cannibalism. Other unaddressed questions are whether emperipolesis is comparable mechanistically to entosis or to cannibalism? Whether cells differentiate between engulfment of another tumour cell or an immune cell?

Larger cancer patient studies, a better understanding of the consequences of chemotherapy on CIC, biomarkers for each CIC formation process as well as the development of automated CIC detection in histological sections of tumour samples are required to use CIC presence as a diagnostic tool in the clinic in the future.

## Abbreviations

CIC: cell-in-cell
WT: wild type
